# An Overview of X-ray Photon Counting Spectral Imaging (x-CSI) with a Focus on Gold Nanoparticle Quantification in Oncology

**DOI:** 10.3390/jimaging8010004

**Published:** 2021-12-31

**Authors:** Oliver L. P. Pickford Scienti, Dimitra G. Darambara

**Affiliations:** Joint Department of Physics, Institute of Cancer Research and Royal Marsden NHS Foundation Trust, London SM2 5NG, UK; dimitra.darambara@icr.ac.uk

**Keywords:** energy resolving, photon counting, spectral imaging, gold nanoparticles, radiotherapy dose enhancement, x-CSI

## Abstract

This review article offers an overview of the differences between traditional energy integrating (EI) X-ray imaging and the new technique of X-ray photon counting spectral imaging (x-CSI). The review is motivated by the need to image gold nanoparticles (AuNP) in vivo if they are to be used clinically to deliver a radiotherapy dose-enhancing effect (RDEE). The aim of this work is to familiarise the reader with x-CSI as a technique and to draw attention to how this technique will need to develop to be of clinical use for the described oncological applications. This article covers the conceptual differences between x-CSI and EI approaches, the advantages of x-CSI, constraints on x-CSI system design, and the achievements of x-CSI in AuNP quantification. The results of the review show there are still approximately two orders of magnitude between the AuNP concentrations used in RDEE applications and the demonstrated detection limits of x-CSI. Two approaches to overcome this were suggested: changing AuNP design or changing x-CSI system design. Optimal system parameters for AuNP detection and general spectral performance as determined by simulation studies were different to those used in the current x-CSI systems, indicating potential gains that may be made with this approach.

## 1. Introduction

Radiotherapy is a staple component of modern cancer therapies due to its ability to be targeted to volumes of interest and to damage cancer cells more readily than their healthy counterparts. The targeting capability in particular allows both for localisation of radiation to tumours that cannot be accessed surgically (e.g., stereotactic radiotherapy for brain tumours) and whole organ coverage to prevent recurrence, without the systemic toxicity associated with chemotherapy (e.g., during whole breast radiation treatment).

The radiotherapy dose that can be delivered to a patient’s tumour is constrained by concerns regarding the effects of incidental radiation dose on healthy tissues. Whilst careful treatment planning and the use of modern multileaf collimators mean that the dose delivered to healthy tissues can be kept many times lower than that delivered to the tumour, concerns regarding side effects and the inducement of secondary tumours limit the radiation dose that can be delivered to tumours. This is particularly problematic in cases where the therapeutic index (relative radiosensitivity of cancer cells to healthy cells) is low, or where radiosensitive healthy structures are located near the treatment site. Therapeutic indices are often reduced where hypoxic regions of the tumour are present [1], and incidental irradiation of nearby structures can cause significant morbidity in several cancer types, including prostate cancers and head and neck cancers [2,3].

**Figure 1:**
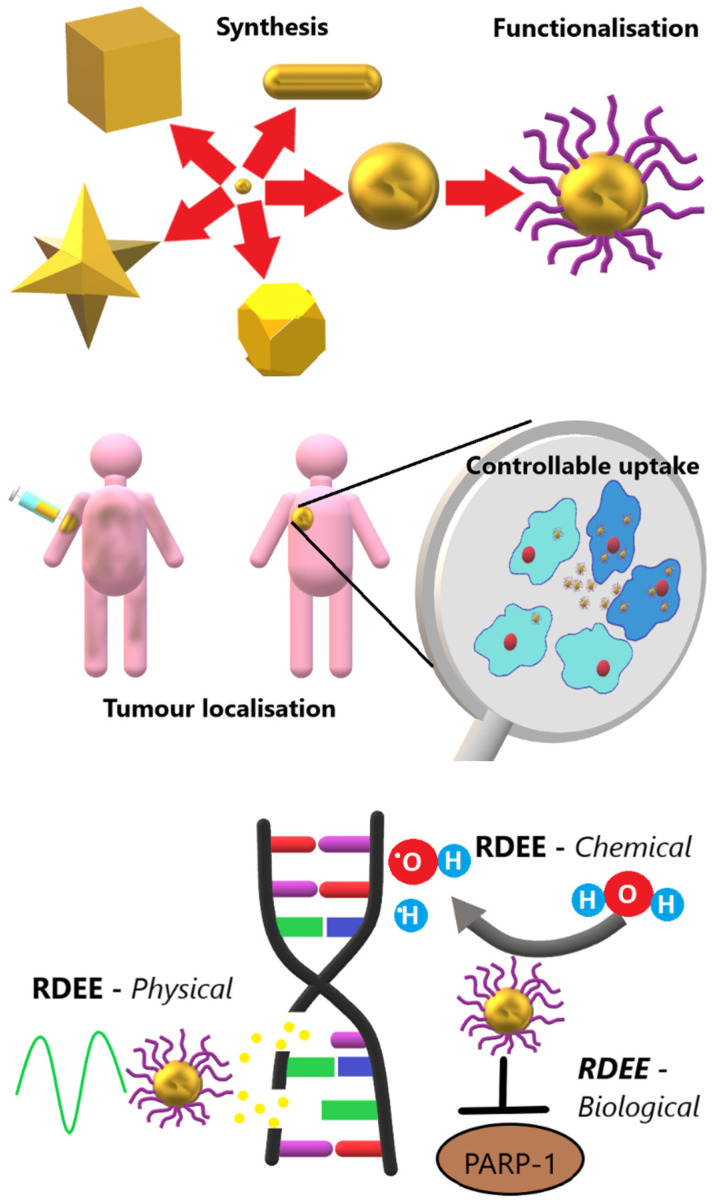
Visual summary of the advantages of AuNPs for delivering RDEE therapeutically. Synthesis: There are multiple shapes that can be synthesised readily and with rudimentary lab supplies. Functionalisation: A range of ligands can be readily attached in combination using click chemistry. Tumour localisation: AuNPs administered intravenously can accumulate preferentially at the site of a tumour. Controllable uptake: AuNPs can be functionalised to enter cancerous cells (darker blue) preferentially, and even targeted to organelles such as the nucleus (red spheres) once internalised. RDEE: Radiotherapy dose-enhancement effects can be delivered by physical (secondary electron generation), chemical (catalytic ROS generation), and biological (DNA repair inhibition) mechanisms. Adapted with permission from ref. [32]. Copyright 2021 The Institute of Cancer Research.

Various approaches have been suggested for improving the therapeutic index by sensitising cancer cells to radiotherapy by reducing hypoxia [4,5], delivering a selective radiosensitizer [6], improving the resistance of healthy cells to radiotherapy using adjuvant drugs [7] or by modulating the time of delivery [8,9]. Additionally, approaches at localising the radiation damage have been proposed, such as the use of ion beam therapies, which deposit their energy over a much smaller region in the patient due to the Bragg peak effect [10]. These approaches can have significant limitations to scaling up, however, such as the cost and technical expertise associated with delivering modern proton therapies. One approach that has been proposed uses gold nanoparticles (AuNP) to combine tumour radiosensitising and radiation localisation approaches at a relatively low cost and without the need for dedicated treatment centres. This approach, which is still at the preclinical stage, involves using the AuNPs to deliver a radiotherapy dose-enhancing effect (RDEE). AuNPs have a range of features that make them suitable for this task, as summarised in Figure 1.

There is extensive literature on tailoring the synthesis of AuNPs to produce a wide range of shapes and sizes [11,12], as well as demonstrations that they can be functionalised to bind different molecular targets [13] and accumulate selectively in cancer cells [14]. A detailed review of the design and use of AuNPs for this purpose is beyond the scope of this review; however, suffice it to say for now that selective targeting has been shown by several groups in limited contexts and that improving the targeting abilities of AuNPs is a matter of ongoing research.

Once accumulated, AuNPs can increase the susceptibility of cancer cells to radiotherapy by delivering a range of different RDEEs. These RDEE mechanisms can be broadly divided into three major categories:

- Physical enhancement mechanisms are those that improve radiotherapy response of cells due to physical interactions between incident radiation and the AuNPs themselves. These can include increased energy deposition (due to the higher Z-number of gold producing a higher probability of interaction with the incident radiation beam) and production of more ionising radiation types when irradiated (production of secondary X-ray fluorescence, Auger electrons, and photoelectrons [15]). These effects have some geometric dependence, but are largely thought to depend primarily on the amount of Au present in the treated cells.
- Chemical enhancement mechanisms are those that improve radiotherapy response of cells due to an increase in the number of reactive oxygen species (ROS) that are produced in the presence of AuNPs compared to in their absence [16]. These effects can be related to interactions between water molecules and the AuNP surface [17,18], and so generally are assumed to depend on the available AuNP surface area within the treated cells.
- Biological enhancement mechanisms are those that alter cell behaviour or metabolism in such a way as to predispose the cells to radiotherapy damage. These range from molecular changes such as inhibition of DNA repair [19], to cell cycle changes that cause accumulation of cells in radiosensitive phases [20]. The mechanisms behind some of these effects are not well understood, though they can reasonably be assumed to relate either to AuNP surface area or the levels of ROS produced by AuNP presence.

It is worth noting that there is much discussion in the literature as to where the line between chemical and biological mechanisms is drawn; however, what is not debated is that the effects mentioned above exist [21,22,23,24]. The wide variety of RDEE mechanisms, combined with their relatively high biocompatibility [25] and long history as a therapeutic agent [11], mean AuNPs have drawn a lot of interest from researchers looking to increase remission rates, though they are equally useful for reducing side effects associated with radiotherapy [26].

Crucially to this review, the use of AuNPs in a clinical setting will require that their distribution within a patient can be imaged and quantified. Initially this ability will be used to confirm the accurate targeting and localisation of AuNPs within tumours or other targets of interest and to refine functionalisation approaches that improve tumour selectivity in humans. In routine clinical practice, quantitative images of AuNPs will again be needed so that the spatial distribution of dose enhancements can be calculated, and the effective dose delivered to organs at risk properly calculated and managed. Whilst the physical properties of AuNPs are often cited as suiting them to optical imaging techniques such as photoacoustic spectral imaging [27,28], it is X-ray imaging techniques that are best placed for clinical use due to their excellent spatial resolution, whole body imaging capability, and sensitivity to the higher Z-number of Au compared with the other human body constituents.

Au seeds are already used as fiducial markers in X-ray imaging [29], and much work already exists on the use of AuNPs as X-ray contrast agents [30,31]. Traditional X-ray techniques struggle to provide reliable and reproducible quantitative images, however, and this has traditionally limited their role to semi-quantitative and structural imaging rather than fully quantitative imaging. Newer developments such as dual energy approaches have corrected for this deficit in some respects; however, shortcomings in noise performance remain, making quantification of the low concentrations relevant to AuNP-mediated RDEE research unlikely. These shortcomings can be overcome, and several new and interesting applications realised, by a move to X-ray photon counting spectral imaging (x-CSI) techniques.

x-CSI approaches are still under development, though clinical systems are available, such as the Extremity 5X120 scanner by MARS Bioimaging [33] and the Naeotom Alpha scanner by Siemens Healthineers, which received FDA approval in September 2021 [34]. In addition, other major medical manufacturers have developed x-CSI system prototypes or announced a move towards commercialisation, such as Phillips Healthcare [30] and GE Healthcare [35]. This review aims to introduce the interested reader to x-CSI techniques, how they differ from conventional X-ray imaging systems, and their potential for use in clinical AuNP-mediated RDEE tasks. A major limitation from a review perspective is the relative sparsity of physical x-CSI systems, as well as the relatively young state of the field. Nevertheless, the recent FDA approval of a photon-counting (PC) system for clinical use makes this review timely in its aim of highlighting the benefits that x-CSI could have in allowing an exciting new clinical technique to be realised.

## 2. Requirements of X-ray Detectors for AuNP Quantification

### 2.1. Scintillators vs. Direct Converters

X-ray imaging works by assessing variations in radiopacity to differentiate different materials and structures. Consider an X-ray beam passing through a biological sample composed of N different materials before impinging on a pixelated X-ray detector. If we ignore scatter and system noise for the moment, the intensity recorded by each pixel, *I*, can be related to a linear summation of the form

(1) $$I\propto \;\frac{1}{\sum_{1}^{n}l_{n}{µ}_{n}}$$

where µ*_n_* is the linear attenuation coefficient of the *n*th component material and *l_n_* is the total path length through that material, traversed by the X-ray beam traveling from source to detector. Importantly, µ*_n_* is a function of incident X-ray energy, and this fact can be used to differentiate otherwise similarly absorbing materials. The X-ray intensity reaching the detector can then be converted into an electrical signal for reading out. Most modern X-ray detectors use either scintillation or direct conversion to perform this conversion.

Scintillators, such as sodium iodide (NaI) or caesium iodide (CsI), convert X-rays into a pulse of photons at visible (or near ultraviolet) wavelengths. These optical photons are then detected and amplified by photomultiplier tubes, or detected using traditional photodiodes, to produce an electrical signal. In contrast, direct converters, such as silicon (Si), gallium arsenide (GaAs), or cadmium telluride (CdTe), convert the absorbed X-ray directly into free charge carriers within the material. These carriers are then collected by applying an electric field across the sensor material to produce the electrical signal measured.

In both approaches, the final electrical signal can be related to the energy of the X-ray photon absorbed in the sensor material. Compared with direct converters, the multiple steps involved in scintillation detectors (optical photon generation, photon multiplication, photon capture and photon to electrical signal conversion) lead to more statistical noise in relating electrical signal and incident X-ray energy. The transition times for each conversion step and variable generation-to-collection path lengths for the optical photons caused by their isotropic emission act to further degrade the temporal and spectral resolution of scintillator approaches in comparison with direct converters. For these reasons, and due to the high temporal resolutions required, PC systems almost exclusively are designed using direct conversion techniques. The remainder of this review will thus concern itself with this type of detection technology.

### 2.2. Photon Counting vs. Energy Integration

Traditional X-ray systems are energy integrating (EI), which differ from PC systems in the way that they use the electrical signals from the sensors to measure X-ray interaction. In general, EI systems integrate the signals generated by multiple X-ray photons to estimate the total X-ray energy deposited in the sensor, whereas PC systems seek to count the number of X-ray photons that are absorbed by the sensor. Each approach has its own advantages and limitations.

#### 2.2.1. Energy Integration

EI systems relate the signal intensity of a given pixel to the total energy deposited in that pixel over the acquisition time, *t_ac_*. Variations between pixels can then be used to determine the differential attenuation of the incident X-ray beam by the material in the source-to-pixel path.

EI approaches have several advantages over the newer photon counting techniques. As they integrate signal over multiple photons, they do not experience detector paralysis, nor do they require particularly fast refresh rates in the electronics, though they should refresh at least as fast as 1/*t_ac_*. Additionally, due to the simpler electronic readouts, lower data outputs (each pixel producing only a single value: integrated charge), and the statistical advantages of averaging shot noise and other random processes over many thousands of photons, EI detectors are comparatively simple and reliable to manufacture consistently and at scale.

Despite these advantages and their widespread use in clinical settings, EI techniques have inherent limitations that constrain future developments. Firstly, we can consider spectral response. As they are only sensitive to total energy deposited, EI systems cannot differentiate the arrival of two photons with energy *E*_1_ and one photon of energy *E*_2_, where *E*_1_ = 2*E*_2_. This introduces a problem in some clinical tasks, as it means that higher-energy photons are overrepresented in the output signal compared with the lower-energy photons. This is particularly problematic for soft tissue discrimination tasks, as their low effective atomic number, *Z_eff_*, means that soft tissues are primarily differentiable in their differing attenuation of lower-energy X-ray photons, being largely transparent to the higher energies used.

**Figure 2:**
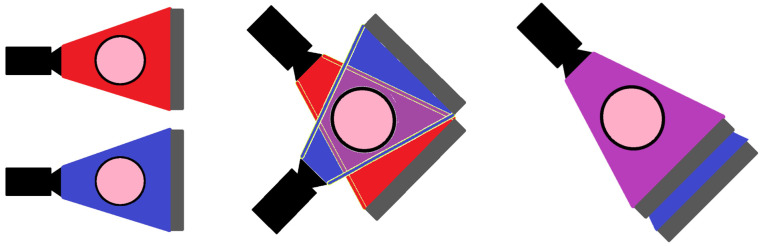
Current dual-energy approaches can be broadly classified into three categories. In all figures, the object being imaged is the pink circle, the X-ray source is in black, the detector is in grey, the high-energy X-ray beam is shown in blue, and the low-energy beam is shown in red. (LEFT) Voltage-switching approaches involve imaging the object twice for each projection angle, once at a lower tube voltage and once at a higher tube voltage. This switching can be performed rapidly so that the resulting images are temporally offset, but spatially approximately coincident. (MIDDLE) Dual-source approaches involve the simultaneous acquisition of the images using two different X-ray beam-detector pairs. The acquired images are thus temporally synced but spatially offset. (RIGHT) Dual-detector approaches involve the use of multiple detectors behind each other. The X-ray beam is attenuated in an energy dependent way as it passes through the first detector such that a beam with a higher average energy spectrum is incident on the second detector. The resulting images are spatially and temporally coincident but show a higher correlation. Adapted with permission from ref. [32]. Copyright 2021 The Institute of Cancer Research.

One approach that can be used to improve soft tissue contrast involves assessing how µ*_n_* varies as a function of X-ray energy. This information can be diagnostically useful, allowing for better delineation of tumours, for example [36]. As they measure the integral of energy deposited in a pixel by all X-ray energies in the incident beam, EI systems are unable to extract spectral information from a single X-ray beam and detector. Multiple approaches exist to overcome this limitation [37], and some of them are illustrated in Figure 2 (adapted with permission from ref. [32]. Copyright 2021 The Institute of Cancer Research). They include rapidly switching the X-ray tube voltage to acquire images at two different X-ray energies per projection [38], using two different X-ray source-detector pairs in each scan [39], or placing a second detector behind the first detector [40] so that the beam seen by the second detector contains a higher proportion of high-energy photons (as lower-energy photons are more likely to be stopped in the first detector than higher-energy ones). Collectively, these are referred to as dual-energy approaches, as they each assess how µ*_n_* differs between two irradiations of differing average X-ray energy. Regardless of the approach chosen, dual-energy EI techniques are associated with inherent temporal/spatial/dose mismatches between the two different acquisitions. This is a fundamental limitation of EI detectors that stems from the fact that each acquisition represents the total energy deposited by multiple photons spanning a range of energies.

Another limitation of EI detectors is the way that noise manifests itself in the final images. Ideally, the pixels in an EI system produce a signal intensity, *I*, which is proportional to the energy deposited in that pixel by incident X-rays. In practice however, external and internal sources of noise can also be summed into the signal, such as light leakage from the external environment (in scintillating detectors) and thermal noise in electronic circuits. This noise is thus superimposed on the collected image. To limit the impact of this noise, higher per-pixel X-ray fluxes are needed so that the signal-to-noise ratio (SNR) is maximised. This can be difficult to achieve in some clinical contexts, such as thicker patient projections or where metal implants may lead to regions of photon starvation [41]. Compensating for the effect of this noise thus sets a minimum radiation dose for a desired image quality. This concern also sets a lower limit on the pixel size used, as smaller pixels will inherently intercept fewer photons at a given X-ray flux.

To overcome these inherent limitations and allow future X-ray technologies to compete more favourably with quantitative molecular imaging modalities such as MRI, a new approach to signal generation has been developed: photon counting.

#### 2.2.2. Photon Counting

**Figure 3:**
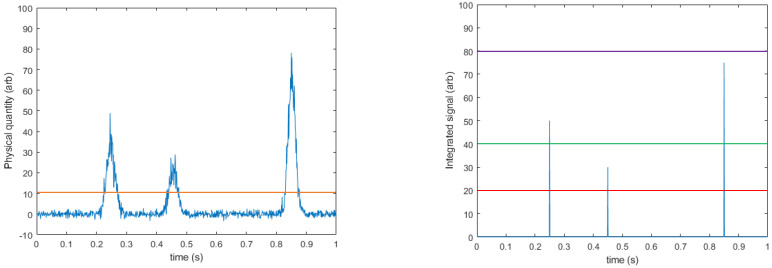
Basic principles behind PC approaches. (LEFT) Some physical feature of the system, such as the charge in a pixel, is shown varying continuously (blue line). Some level of noise is always present in these measurements; however, when a photon interacts with the detector material it causes a sharp and significant change in the measured quantity. A threshold is set (orange line) sufficiently above the noise floor such that any signal rises above this level indicate a photon interaction. A counter is then linked to this threshold and incremented whenever the threshold is crossed from below. The physical quantity then returns to baseline over some time, *dt*. (RIGHT) By using multiple threshold-counter pairs, the energy of each event can be broadly binned. In this example the counter associated with the lowest threshold (red line) would be incremented three times, the middle threshold counter (green line) incremented twice, and the highest-energy threshold counter (purple line) not incremented at all. The number of events between given thresholds is then determined by subtraction of adjacent counters. Adapted with permission from ref. [32]. Copyright 2021 The Institute of Cancer Research.

Photon counting (PC) detectors aim to relate the intensity of a given pixel to the number of photons that interact with that pixel rather than the energy deposited in it. The basics of how this works are summarised in Figure 3 (adapted with permission from ref. [32]. Copyright 2021 The Institute of Cancer Research). Like EI detectors, PC detectors integrate the charge deposited in a pixel by X-rays; however, the integrated charge collected is allowed to bleed off at a carefully calculated speed: slow enough that all of the charge in the pixel can be collected and integrated, regardless of its depth within the pixel, but fast enough that the charge from a single X-ray interaction has completely discharged before a second X-ray interacts with the pixel. Figure 3 shows how the charge on the preamplifier in such a circuit would vary as a function of time where three photons interact with the sensor. The integrated charge is continuously compared with one or more pre-set thresholds within the pixel circuitry. Whenever a threshold is crossed from below, a counter associated with that threshold is incremented to register that a photon of at least that energy has been detected. When the integrated charge decays below that threshold, the system can count again.

The lowest trigger threshold is set sufficiently above the noise floor of the system (at least two standard deviations above the mean of the noise [42]) that it cannot be triggered without the presence of an X-ray interaction. This effectively makes false counts due to electronic noise impossible, and indeed impressive images have been achieved showing zero counts over long exposures when X-ray sources were absent. Compared with EI systems, the elimination of false counts means that even PC systems with only a single threshold can offer a choice between reducing the patient dose for a fixed SNR [43] or improving the SNR for a given patient dose [44]; which approach is taken depends on the clinical context. The removal of noise-induced counts also means that the high per-pixel flux requirements of EI do not apply to PC systems, allowing smaller pixel sizes to be employed, improving spatial resolution in a given source-detector geometry. Furthermore, the shift from energy deposition to photon counting removes the problem of overweighting higher-energy photons in the output signal that is intrinsic to EI systems, allowing for improved soft tissue contrast with PC techniques.

As noted above, PC systems can employ multiple thresholds per pixel, and by subtracting the counts in adjacent thresholds it is possible to calculate the number of photons detected that had an energy between the thresholds. Using Figure 3 as an example, the photons detected were 25, 50, and 75 keV, and they were compared against thresholds of 20, 40, and 80 keV. The system records three counts above threshold 1 (all photons went above this level), two counts above threshold 2 (50 and 75 keV > 40 keV), and zero counts above threshold 3 (no photons were detected above 80 keV). By subtracting counter 3 from counter 2 we find two photons in the range of 40–80 keV, and by subtracting counter 2 from counter 1 we find that one photon was detected in the range of 20–40 keV. The result of this process is that a coarsely binned energy spectrum can be reconstructed from multi-threshold PC systems using only a single detector and a single irradiation. This is a significant advantage over EI systems and saves the additional cost and complexity associated with dual-energy EI designs (see Figure 2). The binning of a single X-ray source also significantly reduces the spectral overlap between the higher- and lower-energy bins, though some may remain due to imperfect energy deposition and collection, as well as where electronic noise shifts photons near the boundary between two thresholds over the line. Additionally, the ability to readily change the thresholds allows the operator more rapid flexibility in selecting the difference between “low”- and “high”-energy bins without having to change filter materials, anode material, or X-ray tube parameters. Detectors that operate in this way to generate multiple energy bins from a single pixelated sensor can be referred to as x-CSI detectors: X-ray photon Counting Spectral Imaging detectors.

### 2.3. Using Spectral Information

As this review paper is focussed on X-ray imaging for AuNP quantification, it is useful to explore how spectral information can be used to provide this. The measured attenuation, *A*, of a given volume can be defined as

(2) $$A={\left[X\right]}_{1}{µ}_{1}+{\left[X\right]}_{2}{µ}_{2}+{\left[X\right]}_{3}{µ}_{3}+\ldots {\left[X\right]}_{n}{µ}_{n}$$

where [*X*]*_n_* and µ*_n_* are the concentration and the attenuation coefficient, respectively, of the *n*th material comprising that volume. µ*_n_* is a fixed and well-defined function of energy that is unique for each component material: µ*_n_* ≠ µ*_m_* ∀ *m* ≠ *n*. By sampling *A* at *N* different energies, we can construct a series of *N* linear equations with a unique solution, so long as the system is critically or overdetermined (*N* ≥ *n*). In this way, we can quantify the concentrations of *N* materials. Due to their short wavelengths, X-rays interact with individual atoms rather than molecules, and there are significantly more than two elements present in human bodies, with most soft tissues comprising H, N, and O as the major components and bone constituting significant levels of Ca. To overcome this, a set of “basis materials” with different atomic compositions are often defined to allow for a semi-quantitative analysis (for example, the material decomposition of a soft tissue voxel into muscle and fat components). Alternatively, the two channels can be used as fitting parameters to determine *Z_eff_* and *ρ_eff_*, the effective atomic number and effective electron density, respectively. This allows the relative ratio of photoelectric and Compton interactions in the sample to be assessed, which will vary between tissue types [45]. The details of this process are beyond the scope of this review; however, note that whilst dual-energy EI approaches can estimate *Z_eff_* and *ρ_eff_*, for most clinical applications material decomposition is preferred. This is due to the higher mathematical instability associated with determining *ρ_eff_* and *Z_eff_* with the two channels in EI systems that contain significant overlap in the energy spectra used for generating each channel. Material decomposition approaches can also be extended to a third basis material where additional information can be incorporated into the reconstruction as constraints; however, these require task-specific algorithms and are time and computational resource intensive [46].

The estimation of *Z_eff_* and *ρ_eff_* is of great interest in the field of oncology specifically, however, as estimations of dose deposition used routinely in radiotherapy planning require estimates of these parameters for accuracy. For proton therapy in particular, uncertainties in *Z_eff_* and *ρ_eff_* could translate into clinically significant differences in dose profile to the patient given the relatively small volume over which proton beams deposit the bulk of their energy. Where *Z_eff_* and *ρ_eff_* accuracy needs optimising or more than three basis materials are needed, dual-energy EI systems will need replacing with truly spectral (*N* > 2) x-CSI systems. Crucially for this review, quantitative AuNP imaging for informing radiotherapy treatment plans will require both of these.

Though x-CSI systems use spectral information in a similar way to dual-energy EI systems, they gain this information in a different way, which adds significant advantages in both quality and flexibility to scale up. By excluding electronic noise from the counts and dividing a single X-ray beam into multiple bins rather than utilising multiple beams, x-CSI systems produce data sets that are intrinsically less noisy and contain less spectral overlap. This point is perhaps stated overly simplistically, and it should be noted that electronic noise is still present in x-CSI systems—it just manifests in a less damaging way (as a small uncertainty in photon energy rather than a large uncertainty in photon counts). Spectral overlap also still occurs in x-CSI systems, due to noise sources, partial energy deposition, and charge-sharing effects [47]. Nevertheless, these problems are less detrimental to x-CSI systems than their counterparts in EI systems for the reasons discussed above. Binning a single X-ray beam also means that *N* can be scaled (relatively) simply by increasing the number of thresholds used in the electronics, without the cost of adding additional sources/detectors or the decrease in temporal resolution associated with additional voltage switches.

It is hard to overstate the transformative potential of x-CSI over dual-energy EI for medical-imaging applications. x-CSI systems are intrinsically more versatile than their EI counterparts, and allow for new applications such as: simultaneous, multi-contrast agent decomposition [48]; improved metal artefact reduction [49,50]; higher-quality imaging of implanted stents [51]; and even quantitative in vivo imaging [52], which is required for the RDEE applications relevant to this review. Lower patient doses, improved soft tissue contrast [53], and the ability to produce quantitative, molecular images [54,55] mean that x-CSI can perform comparably to MRI in a range of applications without the associated costs and logistics associated with maintaining supercooled magnets. x-CSI is thus well placed to ease the pressures on MRI services and to act as a replacement for them in cases where MRI is either unavailable or unsuitable for the patient, due to claustrophobia or metal implants, for example. Despite their promise, x-CSI systems are not without their own drawbacks and limitations, both technical and intrinsic. The remainder of this review article will focus on the promised and demonstrated advantages of x-CSI to date, the outstanding limitations/difficulties, and ongoing work towards developing these systems to overcome them. In all cases, the desired application of x-CSI for facilitating AuNP-mediated RDEE will be kept central.

## 3. Advantages of x-CSI for In Vivo AuNP Imaging

### 3.1. X-ray Imaging vs. Other Imaging Modalities

X-ray imaging techniques have obvious advantages over other clinically available imaging modalities when it comes to imaging nanoparticles (NP). Compared with ultrasound or photoacoustic imaging, X-ray images are easily produced for whole patient volumes, regardless of depth, with less noise and less inter-user variability. Compared with positron emission tomography (PET) (or single photon emission computed tomography (SPECT) for radiolabelled NPs), X-ray scanners provide higher spatial resolution, faster imaging times, and lower ionising radiation doses to patients, and do not require specialist isotope production and nuclear chemistry. Compared with MRI, X-ray imaging is faster, less claustrophobic for the patient, cheaper, and suitable for patients with metal implants and/or cardiac pacemakers.

### 3.2. x-CSI vs. Traditional X-ray Imaging Techniques

Conventional X-ray imaging techniques are of limited use for in vivo AuNP imaging, however, due to a range of outstanding issues. These include blurring and streaking artefacts associated with metal implants/foreign bodies [56]; poor soft tissue contrast, especially in the more commonly available single-energy computed tomography (CT) systems; a high level of electronic noise (compared with the signal size of the low concentrations of AuNPs being looked at); and, significantly, a lack of molecular imaging [54]. Traditional monoenergetic CT systems use Hounsfield units (HU) to score the radiopacity of given pixels/voxels, where HU is defined as

(3) $$\mathrm{HU}=\frac{µ-{µ}_{w}}{{µ}_{w}-{µ}_{a}}$$

In this equation, µ*_w_* is the linear attenuation coefficient for water with respect to the whole monochromatic beam, and µ*_a_* is the linear attenuation coefficient of the beam by air. HU is thus semiquantitative in nature, due to it being fixed by these two points; however, the HU values for various tissue types can vary significantly, even on the same machine, depending on the scanning protocol used (tube energy, filter material and thickness, reconstruction algorithm employed, etc.) [57]. The above variables, combined with biological variations between patients, mean that HU scores for a given tissue type can vary by up to 200 HU between different scans. This is not a problem for structural imaging applications that rely on the relative scores of different tissues (e.g., bones are more radio-dense than soft tissues); however, it complicates the task of quantitative imaging. This is particularly problematic given the high sensitivity required for quantifying the low concentrations of AuNPs demonstrated to produce RDEE. Switching to x-CSI from traditional EI techniques can significantly reduce, or even eliminate, these difficulties.

Iterative metal artefact reduction techniques that account for the spectral information from x-CSI provide unprecedented performance, allowing previously difficult tasks to be achieved with confidence, such as assessing in 3D the state of bone–metal interfaces for surgical screws or other metal implants [51,56]. As noted previously, x-CSI allows for a flexible weighting of “high”- and “low”-energy X-rays, improving soft tissue contrast [58].

The use of higher values of *N* (up to eight in the latest generation of CERN’s Medipix chipset, Medipix3RX [59]) allows for more basis materials to be defined, providing a wider range of soft tissue categories [55] and the ability to simultaneously discriminate tissues and several contrast agents. A particularly powerful demonstration of the potential of x-CSI for this kind of imaging can be seen in the work of MARS Bioimaging, who have used an x-CSI-based small-animal-imaging system to simultaneously image three biological basis materials (bone, lipids, and water) along with three contrast agents in a phantom study [60], and achieved similar results in a mouse model [61]. A similar approach was demonstrated by Siemens Healthineers using a clinical scanner modified for x-CSI (by replacing one of the two EI detectors in a dual-source CT system with a custom PC detector) [48]. This system was used to simultaneously image the kinetics of three high-*Z*-number contrast agents passing through a canine model, though with poorer tissue discrimination and a noisier reconstruction method. These applications reveal an additional advantage of x-CSI: multiple contrast agent scans can be performed at once, reducing patient dose, freeing hospital resources, and removing the problem of image registration compared with serial dual-energy scans.

The additional basis materials can also feasibly be used to enable molecular imaging capabilities in x-CSI by utilising functionalised high-*Z*-number NPs [62]. This would allow the distribution of molecules of interest to be ascertained by proxy [54,63,64].

The lower noise of the PC approach means that x-CSI is a more dose-efficient technique than EI. As a result, x-CSI images can be produced with similar quality but at a fraction of the dose to the patient (67% and 83% dose reductions for sinus and temporal bone examinations, respectively [43]). Whilst lower radiation doses are of primary interest when using dose enhancers such as AuNPs, it is also worth noting that x-CSI could instead produce diagnostically superior images at the same dose as EI techniques (12.8%–40.0% improvement [44,65,66]), though the exact dose savings vary by imaging tasks and have not yet been assessed for AuNP quantification specifically. Nevertheless, should the sensitivity of the systems require improving to detect the exceptionally low Au concentrations associated with AuNP-mediated RDEE, this is a potential possibility without increasing the ionising radiation dose above that already used in EI CT systems.

Finally, the exclusion of false counts from electronic noise allows x-CSI to use smaller pixel sizes than EI CT systems for a given SNR. x-CSI systems can thus produce higher spatial resolutions for a given scanner geometry.

## 4. x-CSI-Specific Design Considerations/Limitations and Their Mitigation

As already noted, x-CSI shares many disadvantages in common with other X-ray imaging techniques, though to a reduced extent in all cases. This section will focus on the considerations specific to x-CSI that limit the applications it is suitable for and the designs that can be achieved.

As x-CSI systems attempt to determine the energy of each incident photon, they require a way to distinguish between different photons interacting with the same pixel in quick succession. Assuming that photon interactions with a pixel are separated by some short time interval, *dt*, this could be achieved by ensuring the time required for the circuitry to collect the charge, increment any counters, and then reset, *dr*, is less than *dt*. This would be a trivial problem to solve if photon interactions were uniformly distributed in time; however, photon interactions are governed by Poisson statistics. This means that the probability of two photons arriving in the same pixel before the circuit can count and reset is non-zero no matter how small *dr* is. For a fixed X-ray flux, the proportion of events for which *dt* < *dr* gets smaller as *dr* is reduced. A minimum value of *dr* is set by physical constraints and electronics parameters. Sufficient time needs to be allowed for the charge carriers in the pixel to be completely collected, regardless of the depth of interaction, to avoid ballistic deficits that degrade the energy resolution of the system. Higher operational voltages can be employed to speed up the collection of charge from the pixel volume; however, there are limits to the voltages that can be used. The proportion of events for which *dt* < *dr* can also be reduced by decreasing the pixel size, increasing *dt* by reducing the per-pixel X-ray flux for a given per-unit-area X-ray flux. Both approaches are used in prototype x-CSI systems, with counting times of the order of tens of nanoseconds and pixel sizes down as far as 50 µm [67,68].

Despite these efforts, keeping *dt* < *dr* for the majority of events places an upper limit on the per-pixel X-ray fluxes that can be used before spectral distortions from pulse pileup or lost counts due to detector paralysis become significant [57]. This limit will depend on both electronic parameters (voltage, shaping time, signal-processing time, charge-sharing correction approaches) and physical parameters (sensor thickness, pixel pitch, presence of anti-scatter grid, etc.), making x-CSI system optimisation for a given spectral imaging task a non-trivial process. Limits on X-ray flux translate into limits on temporal resolution, as X-ray flux cannot be arbitrarily increased to achieve sufficient count statistics in a shorter time. This is in stark contrast with EI systems that benefit from lower system noise when using shorter, higher flux exposures. This makes x-CSI less suitable than dual-energy EI for high flux/extremely fast imaging tasks such as cardiac CT.

A second limitation of x-CSI systems arises from the way in which they separate “low”- and “high“-energy data sets. As only a single beam is used, the “low”- and “high”-energy photons are spatially and temporally coincident on the same pixels, increasing the risk of cross talk between the two. Two issues may arise in this setup: down-counting (where a higher-energy photon is counted in a lower-energy bin) or up-counting (where lower-energy photons are counted in a higher-energy bin). The extent to which each issue occurs depends on pixel size, with larger pixels favouring up-counting (due to multiple photons being summed together) and smaller pixels favouring down-counting (due to the photon depositing only part of its energy within a given pixel’s volume).

Even in the case where a single photon deposits all of its energy within a single pixel volume and no additional photons are present within the count and reset time of the system, there are no guarantees that the full charge generated by the photon will be collected for analysis in that pixel. This is due to the presence of a range of charge-sharing effects (CSEs) that collectively act to distribute charge deposited in one pixel across neighbouring pixels [69]. Fluorescence X-rays, charge cloud expansion (due to diffusion and electrostatic repulsion), and Compton scattering can all act as CSEs [70], though their relatively short ranges mean that spectral degradation from CSEs is most prominent at small pixel sizes (below a few hundred µm) [47]. Nevertheless, if left uncorrected, CSEs can result in X-ray photons registering at a broad range of different energies other than their actual one [70]. Attempts have been made to compensate for CSEs in electronics so that the spatial resolution advantages of smaller pixels can be maintained without the associated loss of spectral resolution. These approaches usually involve some form of charge-sharing correction algorithm (CSCA), which may be implemented either in the analogue domain prior to thresholding [71,72] or in the digital domain post thresholding [73,74].

Pre-thresholding CSCAs involve linking neighbouring pixels together so that CSEs can be identified and corrected for. CSEs are identified where two events are detected in adjacent pixels within some small time window, *dw*. The assumption is made that *dw* << *dt*, such that the probability of two unrelated photons being detected in adjacent pixels within *dw* is negligible and so where this occurs it must be the result of a single photon interaction being shared across multiple pixels. The correction then applied can be either preservative or reconstructive. Preservative CSCAs simply remove the suspected CSE events from the system (prevent associated counters from incrementing) so that, ideally, CSEs are removed from the spectra. This is the simplest correction, though it does result in reduced dose efficiency, as counts are thrown away. In contrast, reconstructive CSCAs attempt to reconstruct the original photon energy by summing the charge found across the involved pixels before thresholding and incrementing counters as normal. Despite the involvement of multiple pixels, only a single pixel’s counters are incremented: usually the pixel with the highest share of the detected charge. Whilst this approach preserves the counts that would otherwise be lost, the reconstructed events may be shifted in energy slightly due to the additive effect of electronic noise from the various pixels.

Pre-thresholding CSCAs can provide impressive gains in spectral resolution at small pixel sizes, though the requirement that *dw* << *dt* limits the acceptable count rates in a similar way to using larger pixels [75]. Selection of a CSCA for a given task is a non-trivial process, with poor CSCA selection able to significantly decrease performance when compared with the uncorrected case. Detailed simulation studies in which CSCAs were parameterised by neighbourhood size, neighbourhood localisation methods, and a reconstruction approach have been conducted that show that the optimal CSCA depends on a range of physical parameters, such as the pixel pitch and the sensor thickness, as well as the intended X-ray flux at which the system will be operated [76]. These simulations also revealed that whilst a CSCA that groups *P* × *P* pixels of size *Y* µm × *Y* µm can maintain the spatial resolution of *Y* µm × *Y* µm pixels, its spectral performance is not as good as if the pixels were replaced with a single pixel of size *PY* µm × *PY* µm and no CSCA. This is due to a range of effects, including charge trapping in inter-anode streets [42] and poorer charge induction efficiencies in the volumes above these streets.

Post-thresholding CSCAs, though well studied [73,74], are employed less often in physical systems. They also identify CSEs based on the close detection times in adjacent pixels; however, rather than correct for the detected sharing pre-thresholding they instead record that a CSE occurred, as well as the pixels involved, using a dedicated counter. This information can then be used to apply corrections after the acquisition has finished. These approaches have several advantages, including simpler electronic implementation, less electronic noise summing, and allowing systems to maintain their temporal resolution. The reduction in spectral distortions is not as significant as with pre-thresholding techniques, however, though these CSCAs represent an interesting approach and may find utility in some applications [73].

Whilst optimising pixel pitch and sensor thickness can be achieved relatively easily with standard simulation tools for other modalities [77,78], the additional complexity associated with x-CSI, including the competing pressures on pixel size for improving spectral resolution and operating flux and the need to select an optimal CSCA, makes x-CSI optimisation more complicated than its EI counterparts. Nonetheless, several groups have modelled x-CSI detectors or selected parts of their imaging chains in an attempt to identify the best design parameters for an x-CSI system [42,79,80,81,82], and our group has done extensive work on optimising x-CSI scanners, including dedicated optimisations for AuNP imaging [47,76,80]. Based on the results so far, it seems likely that rather than a single optimised design, a range of x-CSI scanners will be developed, optimised for maximising spatial resolution, temporal resolution, or spectral resolution, depending on the clinical task.

A final x-CSI-specific concern to note before closing this section involves data storage and management. The detailed images produced by x-CSI are significantly larger than those produced by their competitors. A whole body x-CSI dataset could be ~1 TB in size, compared with DICOM files of hospital CTs, which are usually measured in tens of MB in size [83], and even the most detailed whole body CT dataset publicly available is only ~40 GB [84]. Storage, transfer, and curation of such large datasets will need to be addressed by improvements in healthcare data infrastructures and may be difficult in more remote areas.

x-CSI is clearly more complicated to develop than EI; however, its lower imaging noise, superior soft tissue contrast, and broader spectral capabilities make it an area of great interest to the scientific community. In particular, the next section will look at the potential of x-CSI in implementing AuNP-mediated RDEE by reviewing achievements in AuNP quantification with x-CSI.

## 5. AuNP Quantification with x-CSI

### 5.1. Requirements

Given the specific focus of this review, it is helpful to understand the sensitivities desired of an x-CSI system if it is to be of use in clinical AuNP-mediated RDEE tasks. Comparisons of AuNP concentrations used in imaging and RDEE applications are not simple, in no small part due to the inconsistencies in reporting concentrations found in the AuNP-mediated RDEE literature. Units of mass percentage (mg/kg) [85], mass concentration (mg/mL) [86], particle density (M) [87], and optical density [88] have all been used by various authors. Crucially, though the common unit of molarity makes sense for tracking biological processes that depend on the number of AuNPs (such as cellular uptake), it is not an appropriate unit for use with X-ray imaging techniques. This is because molarity is correctly defined as particles per litre, meaning that studies that use different AuNP shapes/sizes may be using different numbers of Au atoms even if they report using the same molarity. As x-CSI, like all X-ray imaging techniques, is sensitive to Au atom concentration rather than AuNP concentration, this metric may cause confusion.

Table 1 shows the various studies identified from the literature that establish the existence of AuNP-mediated RDEEs, along with both their reported concentrations and a calculated “imaging density,” reported in mg/mL of Au atoms, as this is a metric more commonly used in reporting x-CSI system sensitivities [54,63,64]. The conversion to imaging density was done by:

1. Calculating the volume of the unit cell [89] that is taken up by a single gold atom;
2. Determining the volume of AuNPs used in the study;
3. Dividing the answer from 2 by the answer from 1 to calculate the number of Au atoms per AuNP;
4. Multiplying the answer from 3 by the reported molarities of AuNPs (n.b. the molarity may need to be calculated based on other reported metrics first) to determine the effective number density of Au atoms;
5. Multiplying the answer from 4 by the atomic mass of Au (197) to yield the Au atom density in g/L (which is equivalent to mg/mL).

The above steps lead to the standardisation equation

(4) $$\rho_{i}=197M_{NP}\frac{\frac{4}{3}\pi r_{NP}{}^{3}}{0.017}$$

where *ρ_i_* is the imaging density, *M_NP_* is the molarity of AuNPs used, and *r_NP_* is the radius of the AuNP used. This equation is suitable for nanospheres; however, similar equations can be derived for other nano shapes where needed.

**Table 1:** A summary of AuNP-mediated RDEE studies in the literature. AuNP concentrations reported are given, as well as the calculated: imaging density.” Table adapted with permission from ref. [32]. Copyright 2021 The Institute of Cancer Research.

AuNP Diameter (nm)	Reported AuNP Concentration	Standardised Density (mg/mL)	Reference
1.9	2.4 µM	1.0 × 10^−1^	[87]
1.9	0.24 µM	1.0 × 10^−2^	
13	10 nM	1.3 × 10^−1^	[90]
30	2.4 mg/mL	2.4	[86]
??	36 µg/mL	3.6 × 10^−2^	[91]
14	7 × 10^9^ NPs/mL	1.9 × 10^−4^	[92]
50	7 × 10^9^ NPs/mL	8.8 × 10^−3^	
74	7 × 10^9^ NPs/mL	2.9 × 10^−2^	
1.9	12 µM/500 µg/mL	5.0 × 10^−1^	[93]
2.7	0.5 mg/mL	5.0 × 10^−1^	[94]
14	1.25 nM	2.1 × 10^−2^	[95]
14	2.5 nM	4.2 × 10^−2^	
14	5 nM	8.3 × 10^−2^	
1.9	12 µM/500 µg/mL	5.0 × 10^−1^	[96]
1.9	12 µM/500 µg/mL	5.0 × 10^−1^	[97]
12	1 mM	1.0 × 10^4^	[98]
7	5.5 µmol/mL	1.1 × 10^4^	[99]
47	50 µM	3.1 × 10^4^	[100]
10.8	15 µM	1.1 × 10^2^	[101]
6.1	0.4 mM	5.5 × 10^2^	[102]
6.1	1 mM	1.4 × 10^3^	
4.7	500 µM	3.1 × 10^2^	[103]
14.8	1.5 µg/mL	1.5 × 10^−3^	[104]
14.8	15 µg/mL	1.5 × 10^−2^	
1.9	0.25 mM	1.0 × 10	[105]
1.9	0.5 mM	2.1 × 10	
1.9	1 mM	4.2 × 10	
1.9	12 µM/500 µg/mL	5.0 × 10^−1^	[106]
13	20 nM	2.7 × 10^−1^	[107]
16	20 nM	5.0 × 10^−1^	[108]
49	20 nM	1.4 × 10	
30	15 nM	2.5	[109]
<2	50 µg/mL	5.0 × 10^−2^	[85]
10.8	15 nM	1.1 × 10^−1^	[110]
4.8	0.05 mM	3.4 × 10	[111]
12.1	0.05 mM	5.4 × 10^2^	
27.3	0.05 mM	6.2 × 10^3^	
46.6	0.05 mM	3.1 × 10^4^	

This approach assumes that AuNPs share the same unit cell as bulk gold (which, whilst not strictly true for the smallest AuNPs, holds to a first approximation) and that the mean volume of a sample of AuNPs can be related to the volume of an AuNP with a diameter equal to the sample’s mean diameter. The approach was validated using the subset of studies that reported both molarity and mass density values and it was found that the “imaging density” agreed with the expected mass density to within 0.2%.

In constructing Table 1, it was decided to exclude animal studies and focus on in vitro experiments, as the in vivo RDEE studies often reported the details of the injection rather than the concentration of AuNPs that accumulated at the treatment site, making them unsuitable for relating imaged AuNP concentration to RDEE produced [85,98,112]. The lack of quantification verification at the site of the tumour is also seen in in vivo AuNP imaging studies, with qualitative distributions often reported or quantitative values reported but not verified by an independent method. If the results of in vitro studies are to be used to predict RDEE based on quantitative in vivo images, independent verification of the measured AuNP concentration will be needed in future studies.

At first glance, the studies considered indicate that measurable RDEEs have been demonstrated using Au concentrations ranging from tens of g/mL to a few tenths of µg/mL: eight orders of magnitude. The lowest limit reported to be effective [92] may actually be an underestimate of the Au concentration present at the time of RDEE assessment, however. This study involved several incubation steps with fresh AuNP-containing medium being used at each stage. Given the known ability of cells to take up and retain AuNPs, it is therefore possible that the Au concentration present at the time of RDEE assessment was several times that expected based on the medium used. Discounting this result then, AuNPs appear to produce RDEEs at concentrations down to ~10 µg/mL. RDEE studies have pushed to lower concentrations and found no appreciable RDEE, meaning that this can be taken as a reasonable lower limit that x-CSI systems need to detect.

### 5.2. Achievements

The next section of this review will consider the advances made in AuNP imaging with x-CSI to date. It should be noted that an x-CSI system specifically optimised for AuNP detection has not been developed, and so the results considered may well have room for improvement. Nonetheless, it is instructive to compare the achievements and requirements for this particular application so that the extent and nature of the outstanding work can be commented on.

The use of AuNPs as contrast agents in x-CSI is well known and most physical x-CSI systems that exist have been tested using either AuNPs or aqueous Au solutions. Despite this, the relative sparsity of physical x-CSI detectors, let alone of full x-CSI systems, means that the total number of such studies is small. For this review, we will also consider studies performed on dual-energy PC systems more generally, as these are reasonably expected to perform close, but not superior to, full x-CSI systems. These results will therefore contribute to establishing minimum quantification capabilities that x-CSI can be expected to have when fully developed.

Work by the MARS group using their pre-clinical small-animal-imaging scanner has shown that x-CSI techniques can distinguish contrast agents with as little as 4 keV between their K-edges [61]. They were further able to quantify AuNP concentrations in phantom studies as low as 2 mg/mL with relative ease [64]. This scanner was based on CERN’s Medipix3RX application specific integrated circuit (ASIC) and their scans were performed using the CSCA employed in this chipset and four energy thresholds (though the ASIC can support up to eight different energy bins in other configurations). The study involved imaging serial dilutions of aqueous AuNP solutions, and it should be noted that 2 mg/mL was not shown to be a limiting concentration for the technique but rather represents the lowest concentration investigated. Importantly, the same scanner was able to detect 0.15 µg of AuNP in a cell pellet with relatively low uncertainty; however, the size of the pellet was not reported and so an assessment in terms of concentration cannot be made. Given that the cell pellet was contained in a 0.5 mL Eppendorf tube, however, this sets a lower limit for the concentration detected of 0.3 µg/mL. So long as the pellet took up no less than 1/3 of the Eppendorf tube’s volume, this would be the lowest concentration of AuNPs quantified to date. Unfortunately, the absence of a reported cell pellet volume and verification of AuNP content by a recognised quantitative technique (e.g., ICP-MS, ICP-OES or AES) means this study cannot confirm detection below 2 mg/mL at this time.

AuNP concentrations down to 1 mg/mL have been reported by other groups using x-CSI systems based on modified Phillips Healthcare scanners [30]. This study also used a serial dilution of AuNP solutions to calibrate the scanner, and again, 1 mg/mL was simply the lowest concentration considered. This scanner used a small field-of-view (168 mm) and a standard clinical X-ray tube (filtered, 120 kVp), and the x-CSI detector had five configurable thresholds. The calibrated scanner was then used to produce quantitative images of AuNPs in vivo in a mouse model. These images indicated AuNP concentrations ranging from < 1–5 mg/mL of Au, though the lower concentrations were associated with significant uncertainties in quantification (~1 mg/mL). Importantly, AuNP quantifications in imaged organs were compared with ex vivo analysis of the organs using ICP-OES. The ICP-OES results showed that whilst good linearity in the correlation between ICP-OES and x-CSI quantifications existed, x-CSI quantification systematically underestimated the Au content found by ICP-OES by ~23%. For this reason, the results of this study should cautiously be interpreted as demonstrating that Au concentrations of at least ~1 mg/mL are detectable using current x-CSI techniques. This compares favourably with other cutting-edge X-ray-based imaging and quantification techniques such as X-ray fluorescence computed tomography (XFCT), which has shown similar detection limits [113,114]. Though it is believed that XFCT can improve sensitivity by an order of magnitude [115], x-CSI would remain the imaging modality of choice for this particular application given the advantages of x-CSI in terms of superior imaging depths, improved dose efficiency, and improved soft tissue imaging.

It is apparent that detection and quantification of AuNP concentrations as low as a few mg/mL have been achieved with x-CSI systems. Crucially, this has been shown to be possible in vivo, but the relevant question to this review is how these concentrations compare with the AuNP concentrations that will likely be used in AuNP-mediated RDEE therapies. Table 1 indicates that the concentrations detected are still two orders of magnitude above those known to induce RDEE. This is not to say that x-CSI is not a useful tool for in vivo AuNP quantification for this task however, as ~58% of the RDEE studies considered here used AuNP concentrations that would be quantifiable with existing x-CSI systems. Clinically, the detection limits should include all concentrations that can induce RDEE or toxicity so that the effects of treatment can be accurately modelled; however, there is more than one way to achieve this.

AuNPs could be modified to reduce their toxicity/RDEE efficacy so that they do not produce appreciable effects at concentrations below those detectable with x-CSI. Chitthrani et al. [92] demonstrated a dependence of cellular uptake (and consequently RDEE) on AuNP size: AuNPs which required excessive curvature (AuNPs too small) or excessive resources (AUNPs too big) to endocytose experienced lower overall uptake, leaving an optimal size (~50 nm) for AuNP uptake. Though the data in that paper were provided to support a different point, examination of the figures provided indicated that the AuNPs used did not produce appreciable RDEEs at concentrations below ~10^7^ NPs/mL (~1.7 × 10^−5^ mg/mL when standardised as above). These data thus support the claim that RDEE effects are negligible below some minimum AuNP concentration, implying a lower detection limit needed for AuNP imaging for AuNP-mediated RDEE applications. Whilst it is reasonably inferred that AuNP design should affect this limit due to the many design parameters known to affect RDEE [116,117], variations in lower limits are not known, possibly due to a reluctance to publish negative results in the literature [118]. Furthermore, the relative contributions of the various RDEE mechanisms are not understood well enough at the moment for the task of designing AuNPs with a specified minimum RDEE concentration to be achieved based on much more than trial and error [21,23,119,120], and both RDEE and toxicity are known to vary quite significantly based on the surface coating and cell type in question [121,122,123]. Nevertheless, were the resources invested, this approach may have merit.

An alternative approach is to improve the detection limits of x-CSI systems so that they can quantify the lower AuNP concentrations already used in the literature. Whilst it is true that the studies mentioned have not tried to push the detection limits lower, the uncertainties associated with the images that were obtained indicate these systems would struggle to accurately quantify AuNPs at the lowest concentrations reviewed. No x-CSI system to date has been specifically designed and optimised for the task of AuNP imaging, however. Simulation studies to determine optimal imaging parameters for this task have been performed [47,76], and the optimal parameters suggested for this task (pixel pitch of 200 µm–250 µm, sensor thickness of ~1.5 mm, dynamic Hybrid/3 × 3 type CSCA [32,47,76]) differ from those used in the aforementioned studies (Table 2), hinting at the possibility that gains in performance can indeed be achieved by modified hardware. The values suggested in this simulation study were in good agreement with other optimisation-focused x-CSI simulations and differ from the majority of systems in development at the moment [58], supporting the assertion that current x-CSI scanner performance can be improved. Furthermore, the development of AuNP-specific reconstruction algorithms or imaging protocols could also assist in closing the gap between the current sensitivity and that needed for AuNP-mediated RDEE applications.

The technical requirements associated with producing such an optimised system are not significant given pixel pitches and sensor thicknesses both smaller and larger than the optimal ones are used already, and the CSCA parameters are already combined in some ASICS. Whilst the AuNP detection limits of such an optimised system have not yet been calculated, it is reasonable to assume based on the weight of the literature that at least an order of magnitude increase in sensitivity can be made.

Though a gap of two orders of magnitude is a lot of ground to cover, the two approaches mentioned above are worth exploring, and it may be that clinical realisation initially requires a combination of them both in the short term, or the use of locally introduced and relatively immobile AuNPs so that an assumption can be made as to the maximum distribution of the AuNPs based on their initial placement. Such local immobilisation would limit some of the selective uptake benefits of AuNPs, but would allow for the collection of valuable data on how AuNP concentration and RDEE delivered correlate.

## 6. Summary

The use of AuNPs to enhance the effectiveness of radiotherapy is a well-studied topic, though the corollary requirement of quantitative in vivo AuNP imaging is often taken for granted in the RDEE literature. This review introduced x-CSI techniques as a way to achieve this imaging and explained the fundamental differences between them and more conventional X-ray-imaging approaches.

Whilst it is widely recognised that AuNPs offer an exciting new imaging contrast for x-CSI systems, promising molecular-imaging capabilities, the AuNP concentrations tested in practical systems (~1 mg/mL) are orders of magnitude higher than those likely to produce clinical RDEE effects. For the specific task of AuNP-mediated RDEE delivery, current x-CSI scanners are thus not yet suitable.

Two paths for closing this sensitivity gap are proposed: modifying AuNPs to be less-effective RDEE agents at low concentration and optimising x-CSI scanners for AuNP detection. Specific advice from this review included the more regular reporting of negative results in RDEE literature (so that lower limits on AuNP concentration needed to induce appreciable RDEE can be established) and that x-CSI systems in future be tested using Au concentrations that overlap more significantly with those used in the RDEE literature, so that lower limits of x-CSI systems can be determined directly rather than inferred from extrapolation of data at much higher concentrations.

The intrinsic trade-offs in optimising an x-CSI system for different tasks mean that instead of a single general-purpose scanner, task-specific scanners may be more common. It is hoped that the recent approval of a commercial x-CSI system by the FDA signals the first of many developments in these scanners, and that the high research interest in Au as a contrast agent is translated into a scanner optimised for Au imaging in the not-too-distant future.

## Figures and Tables

**Table 2:** Comparison of values for several key design parameters, as used in leading detectors and a simulation-optimised system. Charge-sharing correction algorithm (CSCA) parameters of neighbourhood size (NS) and neighbourhood location (NL) are looked at here and defined in other publications [76].

	Optimised Detector	MARS Detector	Phillips Healthcare Detector
Pixel pitch (µm)	200–250	110	500
Sensor thickness (mm)	~1.5	2	2
CSCA NS	3 × 3 or Hybrid	Hybrid	1 × 1 or 2 × 2 (chequered)
CSCA NL	Dynamic	Dynamic	Static

## Data Availability

Not applicable.

## References

[1] Moeller B.J., Richardson R.A., Dewhirst M.W. (2007). Hypoxia and radiotherapy: Opportunities for improved outcomes in cancer treatment. Cancer Metastasis Rev..

[2] Elliott S.P., Malaeb B.S. (2010). Long-term urinary adverse effects of pelvic radiotherapy. World J. Urol..

[3] Tolentino E., Centurion B.S., Ferreira L.H.C., De Souza A.P., Damante J.H., Rubira-Bullen I.R. (2011). Oral adverse effects of head and neck radiotherapy: Literature review and suggestion of a clinical oral care guideline for irradiated patients. J. Appl. Oral Sci..

[4] Nimalasena S., Gothard L., Anbalagan S., Allen S., Sinnett V., Mohammed K., Kothari G., Musallam A., Lucy C., Yu S. (2020). Intratumoral Hydrogen Peroxide with Radiation Therapy in Locally Advanced Breast Cancer: Results from a Phase 1 Clinical Trial. Int. J. Radiat. Oncol..

[5] Ho Y.-J., Chu S.-W., Liao E.-C., Fan C.-H., Chan H.-L., Wei K.-C., Yeh C.-K. (2019). Normalization of Tumor Vasculature by Oxygen Microbubbles with Ultrasound. Theranostics.

[6] Chen Y., Gao P., Wu T., Pan W., Li N., Tang B. (2020). Organelle-localized radiosensitizers. Chem. Commun..

[7] Johnke R.M., Sattler J.A., Allison R.R. (2014). Radioprotective agents for radiation therapy: Future trends. Future Oncol..

[8] Guo P., Wang H., Jiang R., Wang Z. (2014). The clinical effect study on malignant tumors with chronoradiotherapy. Biol. Rhythm. Res..

[9] Greco C., Pares O., Pimentel N., Louro V., Santiago I., Vieira S., Stroom J., Mateus D., Soares A., Marques J. (2021). Safety and Efficacy of Virtual Prostatectomy with Single-Dose Radiotherapy in Patients with Intermediate-Risk Prostate Cancer. JAMA Oncol..

[10] Nichelatti E., Ronsivalle C., Picardi L., Montereali R.M. Optimization of the theoretical dose distribution in the ‘Spread out Bragg Peak’ (SOBP) region in proton therapy by means of semi-analytical techniques. Proceedings of the Nuovo Cimento della Societa Italiana di Fisica C.

[11] Pricker S.P. (1996). Medical uses of gold compounds: Past, present and future. Gold Bull..

[12] Herizchi R., Abbasi E., Milani M., Akbarzadeh A. (2016). Current methods for synthesis of gold nanoparticles. Artif. Cells Nanomed. Biotechnol..

[13] Goddard Z.R., Marín M.J., Russell D.A., Searcey M. (2020). Active targeting of gold nanoparticles as cancer therapeutics. Chem. Soc. Rev..

[14] Wang C., Bao C., Liang S., Fu H., Wang K., Deng M., Liao Q., Cui D. (2014). RGD-conjugated silica-coated gold nanorods on the surface of carbon nanotubes for targeted photoacoustic imaging of gastric cancer. Nanoscale Res. Lett..

[15] Butterworth K.T., McMahon S.J., Taggart L.E., Prise K.M. (2013). Radiosensitization by gold nanoparticles: Effective at megavoltage energies and potential role of oxidative stress. Transl. Cancer Res..

[16] Howard D., Sebastian S., Le Q.V.-C., Thierry B., Kempson I. (2020). Chemical Mechanisms of Nanoparticle Radiosensitization and Radioprotection: A Review of Structure-Function Relationships Influencing Reactive Oxygen Species. Int. J. Mol. Sci..

[17] Cheng N.N., Starkewolf Z., Davidson R.A., Sharmah A., Lee C., Lien J., Guo T. (2012). Chemical Enhancement by Nanomaterials under X-ray Irradiation. J. Am. Chem. Soc..

[18] Gilles M., Brun E., Sicard-Roselli C. (2018). Quantification of hydroxyl radicals and solvated electrons produced by irradiated gold nanoparticles suggests a crucial role of interfacial water. J. Colloid Interface Sci..

[19] Turnbull T., Douglass M., Williamson N.H., Howard D., Bhardwaj R., Lawrence M., Paterson D.J., Bezak E., Thierry B., Kempson I.M. (2019). Cross-Correlative Single-Cell Analysis Reveals Biological Mechanisms of Nanoparticle Radiosensitization. ACS Nano.

[20] Xu W., Teng L., Pang B., Li P., Chuanqing Z., Huang P., Zhang C., Qiushi R., Wenbin H., Fu S. (2012). The radiosensitization of melanoma cells by gold nanorods irradiated with MV X-ray. Nano Biomed. Eng..

[21] Her S., Jaffray D.A., Allen C. (2017). Gold nanoparticles for applications in cancer radiotherapy: Mechanisms and recent advancements. Adv. Drug Deliv. Rev..

[22] Rosa S., Connolly C., Schettino G., Butterworth K.T., Prise K.M. (2017). Biological mechanisms of gold nanoparticle radiosensitization. Cancer Nanotechnol..

[23] Butterworth K.T., McMahon S.J., Currell F.J., Prise K.M. (2012). Physical basis and biological mechanisms of gold nanoparticle radiosensitization. Nanoscale.

[24] Liu Y., Zhang P., Li F., Jin X., Li J., Chen W., Li Q. (2018). Metal-based NanoEnhancers for Future Radiotherapy: Radiosensitizing and Synergistic Effects on Tumor Cells. Theranostics.

[25] Shukla R., Bansal V., Chaudhary M., Basu A., Bhonde R.R., Sastry M. (2005). Biocompatibility of Gold Nanoparticles and Their Endocytotic Fate Inside the Cellular Compartment: A Microscopic Overview. Langmuir.

[26] Bromma K., Cicon L., Beckham W., Chithrani D.B. (2020). Gold nanoparticle mediated radiation response among key cell components of the tumour microenvironment for the advancement of cancer nanotechnology. Sci. Rep..

[27] Cheheltani R., Ezzibdeh R.M., Chhour P., Pulaparthi K., Kim J., Jurcova M., Hsu J.C., Blundell C., Litt H.I., Ferrari V.A. (2016). Tunable, biodegradable gold nanoparticles as contrast agents for computed tomography and photoacoustic imaging. Biomaterials.

[28] Jiang X., Du B., Tang S., Hsieh J., Zheng J. (2019). Photoacoustic Imaging of Nanoparticle Transport in the Kidneys at High Temporal Resolution. Angew. Chem. Int. Ed..

[29] Joon D.L., Smith D., Tacey M., Schneider M., Harris B., Ong W.L., Foroudi F., Jenkins T., Wada M., Chao M. (2021). A phantom study to contrast and compare polymer and gold fiducial markers in radiotherapy simulation imaging. Sci. Rep..

[30] Si-Mohamed S., Cormode D.P., Bar-Ness D., Sigovan M., Naha P.C., Langlois J.-B., Chalabreysse L., Coulon P., Blevis I., Roessl E. (2017). Evaluation of spectral photon counting computed tomography K-edge imaging for determination of gold nanoparticle biodistribution in vivo. Nanoscale.

[31] Dong Y.C., Hajfathalian M., Maidment P.S.N., Hsu J.C., Naha P.C., Si-Mohamed S., Breuilly M., Kim J., Chhour P., Douek P. (2019). Effect of Gold Nanoparticle Size on Their Properties as Contrast Agents for Computed Tomography. Sci. Rep..

[32] Pickford Scienti O. (2021). On the Potential of Multi-Spectral X-ray and Photoacoustic Imaging to Facilitate Gold Nanoparticle Mediated Dose-Enhanced Radiotherapy.

[33] MARS for Clinicians (2020). MARS Bioimaging Limited. https://www.marsbioimaging.com/clinicians/.

[34] Siemens (2021). NAEOTOM Alpha® with Quantum Technology. https://www.siemens-healthineers.com/computed-tomography/photon-counting-ct-scanner/naeotom-alpha.

[35] GE Healthcare (2021). Karolinska Institutet & MedTechLabs Kickoff the World’s First Clinical Evaluation of GE Healthcare’s Photon Counting CT Technology with Deep Silicon Detectors. https://www.ge.com/news/press-releases/karolinska-institutet-medtechlabs-kickoff-the-worlds-first-clinical-evaluation-of-ge#_edn1.

[36] Noid G., Tai A., Liu Y., Li X. (2016). TH-CD-202-03: Enhancing Soft-Tissue CT Contrast for Radiation Therapy Using Mono-Energetic Decompositions of Dual Energy CT. Med. Phys..

[37] Goo H.W., Goo J.M. (2017). Dual-Energy CT: New Horizon in Medical Imaging. Korean J. Radiol..

[38] Matsui K., Machida H., Mitsuhashi T., Omori H., Nakaoka T., Sakura H., Ueno E. (2014). Analysis of coronary arterial calcification components with coronary CT angiography using single-source dual-energy CT with fast tube voltage switching. Int. J. Cardiovasc. Imaging.

[39] Petersilka M., Bruder H., Krauss B., Stierstorfer K., Flohr T.G. (2008). Technical principles of dual source CT. Eur. J. Radiol..

[40] Ananthakrishnan L., Duan X., Xi Y., Lewis M.A., Pearle M.S., Antonelli J.A., Goerne H., Kolitz E.M., Abbara S., Lenkinski R.E. (2018). Dual-layer spectral detector CT: Non-inferiority assessment compared to dual-source dual-energy CT in discriminating uric acid from non-uric acid renal stones ex vivo. Abdom. Radiol..

[41] Mori I., Machida Y., Osanai M., Iinuma K. (2013). Photon starvation artifacts of X-ray CT: Their true cause and a solution. Radiol. Phys. Technol..

[42] Lai X., Shirono J., Araki H., Budden B., Cai L., Kawata G., Miyazaki H., Qiang Y., Ye Z., Zhan X. (2020). Modeling Photon Counting Detector Anode Street Impact on Detector Energy Response. IEEE Trans. Radiat. Plasma Med. Sci..

[43] Rajendran K., Voss B.A., Zhou W., Tao S., Delone D.R., Lane J.I., Weaver J.M., Carlson M.L., Fletcher J.G., McCollough C.H. (2020). Dose Reduction for Sinus and Temporal Bone Imaging Using Photon-Counting Detector CT With an Additional Tin Filter. Investig. Radiol..

[44] Pourmorteza A., Symons R., Reich D., Bagheri M., Cork T., Kappler S., Ulzheimer S., Bluemke D. (2017). Photon-Counting CT of the Brain: In vivo Human Results and Image-Quality Assessment. Am. J. Neuroradiol..

[45] Rebuffel V., Dinten J.-M. (2007). Dual-energy X-ray imaging: Benefits and limits. Insight-Non-Destructive Test. Cond. Monit..

[46] Mendonca P., Lamb P., Sahani D.V. (2014). A Flexible Method for Multi-Material Decomposition of Dual-Energy CT Images. IEEE Trans. Med. Imaging.

[47] Scienti O.L.P.P., Bamber J.C., Darambara D.G. (2020). The effects of spectral X-ray photon counting detector parameters on detector performance: Thickness and pitch. IEEE Access.

[48] Symons R., Krauss B., Sahbaee P., Cork T.E., Lakshmanan M.N., Bluemke D.A., Pourmorteza A. (2017). Photon-counting CT for simultaneous imaging of multiple contrast agents in the abdomen: An in vivo study. Med. Phys..

[49] Nasirudin R.A., Mei K., Panchev P., Fehringer A., Pfeiffer F., Rummeny E.J., Fiebich M., Noël P.B. (2016). Reduction of metal artifact in single photon-counting computed tomography by spectral-driven iterative reconstruction technique. PLoS ONE.

[50] Broeke L.V., Grillon M., Yeung A.W.K., Wu W., Tanaka R., Vardhanabhuti V. (2021). Feasibility of photon-counting spectral CT in dental applications—a comparative qualitative analysis. BDJ Open.

[51] Sigovan M., Si-Mohamed S., Bar-Ness D., Mitchell J., Langlois J.-B., Coulon P., Roessl E., Blevis I., Rokni M., Rioufol G. (2019). Feasibility of improving vascular imaging in the presence of metallic stents using spectral photon counting CT and K-edge imaging. Sci. Rep..

[52] Si-Mohamed S., Tatard-Leitman V., Laugerette A., Sigovan M., Pfeiffer D., Rummeny E.J., Coulon P., Yagil Y., Douek P., Boussel L. (2019). Spectral Photon-Counting Computed Tomography (SPCCT): In-vivo single-acquisition multi-phase liver imaging with a dual contrast agent protocol. Sci. Rep..

[53] Symons R., Pourmorteza A., Sandfort V., Ahlman M.A., Cropper T., Mallek M., Kappler S., Ulzheimer S., Mahesh M., Jones E.C. (2017). Feasibility of Dose-reduced Chest CT with Photon-counting Detectors: Initial Results in Humans. Radiology.

[54] Roeder R.K., Curtis T.E., Nallathamby P.D., Irimata L.E., McGinnity T.L., Cole L.E., Vargo-Gogola T., Cowden Dahl K.D. (2017). Nanoparticle imaging probes for molecular imaging with computed tomography and application to cancer imaging. Medical Imaging 2017: Physics of Medical Imaging.

[55] Aamir R., Chernoglazov A.I., Bateman C., Butler P.H., Anderson N.G., Bell S.T., Panta R.K., Healy J.L., Mohr J.L., Rajendran K. (2014). MARS spectral molecular imaging of lamb tissue: Data collection and image analysis. J. Instrum..

[56] Amma M.R., Butler A.P.H., Raja A., Bamford B., Butler P.H., Walker P., Matanaghi A., Adebileje S.A., Anderson N., Anjomrouz M. Assessment of metal implant induced artefacts using photon counting spectral CT. Proceedings of the Developments in X-ray Tomography XII.

[57] DenOtter T.D., Schubert J. (2021). Hounsfield Unit. StatPearls.

[58] Willemink M.J., Persson M., Pourmorteza A., Pelc N.J., Fleischmann D. (2018). Photon-counting CT: Technical Principles and Clinical Prospects. Radiology.

[59] Ballabriga R., Alozy J., Blaj G., Campbell M., Fiederle M., Frojdh E., Heijne E.H.M., Llopart X., Pichotka M.P., Procz S. (2013). The Medipix3RX: A high resolution, zero dead-time pixel detector readout chip allowing spectroscopic imaging. J. Instrum..

[60] Moghiseh M., Aamir R., Panta R.K., de Ruiter N.J.A., Chernoglazov A., Healy J., Butler A.P.H., Anderson N.G. (2016). Discrimination of Multiple High-Z Materials by Multi- Energy Spectral CT—A Phantom Study. JSM Biomed. Imaging Data.

[61] Anderson N.G., Butler A.P., Scott N.J.A., Cook N.J., Butzer J.S., Schleich N., Firsching M., Grasset R., De Ruiter N., Campbell M. (2010). Spectroscopic (multi-energy) CT distinguishes iodine and barium contrast material in MICE. Eur. Radiol..

[62] Balegamire J., Vandamme M., Chereul E., Si-Mohamed S., Maache S.A., Almouazen E., Ettouati L., Fessi H., Boussel L., Douek P. (2020). Iodinated polymer nanoparticles as contrast agent for spectral photon counting computed tomography. Biomater. Sci..

[63] Pan D., Schmieder A.H., SenPan A., Yang X., Wickline S.A., Roessl E., Proksa R., Schirra C.O., Lanza G.M. (2017). Molecular Imaging with Spectral CT Nanoprobes. Design and Applications of Nanoparticles in Biomedical Imaging.

[64] Moghiseh M., Lowe C., Lewis J.G., Kumar D., Butler A., Anderson N., Raja A., Bombonati A. (2018). Spectral Photon-Counting Molecular Imaging for Quantification of Monoclonal Antibody-Conjugated Gold Nanoparticles Targeted to Lymphoma and Breast Cancer: An In vitro Study. Contrast Media Mol. Imaging.

[65] Rajagopal J.R., Farhadi F., Solomon J., Sahbaee P., Saboury B., Pritchard W.F., Jones E.C., Samei E. (2020). Comparison of Low Dose Performance of Photon-Counting and Energy Integrating CT. Acad. Radiol..

[66] Zhou W., Lane J., Carlson M., Bruesewitz M., Witte R., Koeller K., Eckel L., Carter R., McCollough C., Leng S. (2018). Comparison of a Photon-Counting-Detector CT with an Energy-Integrating-Detector CT for Temporal Bone Imaging: A Cadaveric Study. Am. J. Neuroradiol..

[67] Otfinowski P., Deptuch G., Maj P. (2020). FRIC—a 50 μm pixel-pitch single photon counting ASIC with Pattern Recognition algorithm in 40 nm CMOS technology. J. Instrum..

[68] Ballabriga R., Alozy J., Bandi F.N., Campbell M., Egidos N., Fernandez-Tenllado J.M., Heijne E.H.M., Kremastiotis I., Llopart X., Madsen B.J. (2021). Photon Counting Detectors for X-ray Imaging with Emphasis on CT. IEEE Trans. Radiat. Plasma Med. Sci..

[69] Flohr T., Petersilka M., Henning A., Ulzheimer S., Ferda J., Schmidt B. (2020). Photon-counting CT review. Phys. Med..

[70] Ponchut C. (2008). Correction of the charge sharing in photon-counting pixel detector data. Nucl. Instrum. Methods Phys. Res. Sect. A Accel. Spectrom. Detect. Assoc. Equip..

[71] Krzyzanowska A., Deptuch G.W., Maj P., Grybos P., Szczygiel R. (2017). Characterization of the Photon Counting CHASE Jr., Chip Built in a 40-nm CMOS Process with a Charge Sharing Correction Algorithm Using a Collimated X-ray Beam. IEEE Trans. Nucl. Sci..

[72] Ballabriga R., Campbell M., Heijne E.H.M., Llopart X., Tlustos L. (2007). The Medipix3 Prototype, a Pixel Readout Chip Working in Single Photon Counting Mode with Improved Spectrometric Performance. IEEE Trans. Nucl. Sci..

[73] Hsieh S.S., Sjolin M. (2018). Digital count summing vs analog charge summing for photon counting detectors: A performance simulation study. Med. Phys..

[74] Hsieh S.S. (2020). Coincidence Counters for Charge Sharing Compensation in Spectroscopic Photon Counting Detectors. IEEE Trans. Med. Imaging.

[75] Weber N., Ullberg C., Eriksson C., Urech M., Stewart A. (2018). Photon counting dual energy X-ray imaging at CT count rates: Measurements and implications of in-pixel charge sharing correction. Medical Imaging 2018: Physics of Medical Imaging.

[76] Scienti O.L.P.P., Bamber J.C., Darambara D.G. (2020). CdTe Based Energy Resolving, X-ray Photon Counting Detector Performance Assessment: The Effects of Charge Sharing Correction Algorithm Choice. Sensors.

[77] Guerra P., Santos A., Darambara D.G. (2009). An investigation of performance characteristics of a pixellated room-temperature semiconductor detector for medical imaging. J. Phys. D Appl. Phys..

[78] Genocchi B., Scienti O.P., Darambara D. (2017). Optimal configuration of a low-dose breast-specific gamma camera based on semiconductor CdZnTe pixelated detectors. J. Phys. Conf. Ser..

[79] Myronakis M.E., Zvelebil M., Darambara D.G. (2012). Computational modelling of pixelated CdZnTe detectors for x- and γ-ray imaging applications. J. Instrum..

[80] Scienti O.L.P.P., Bamber J.C., Darambara D. (2020). Inclusion of a Charge Sharing Correction Algorithm into an X-ray Photon Counting Spectral Detector Simulation Framework. IEEE Trans. Radiat. Plasma Med. Sci..

[81] Koenig T., Zuber M., Hamann E., Cecilia A., Ballabriga R., Campbell M., Ruat M., Tlustos L., Fauler A., Fiederle M. (2014). How spectroscopic X-ray imaging benefits from inter-pixel communication. Phys. Med. Biol..

[82] Trueb P., Zambon P., Broennimann C. (2017). Assessment of the spectral performance of hybrid photon counting X-ray detectors. Med. Phys..

[83] (2021). A Crash Course on Handling DICOM Medical Imaging Data, POSTDICOM. https://www.postdicom.com/en/blog/handling-dicom-medical-imaging-data.

[84] Ackerman M.J. (1998). The Visible Human Project. Proceedings of the IEEE. https://www.nlm.nih.gov/research/visible/visible_human.html.

[85] Zhang X.D., Chen J., Luo Z., Wu D., Shen X., Song S.-S., Sun Y.-M., Liu P.-X., Zhao J., Huo S. (2013). Enhanced Tumor Accumulation of Sub-2 nm Gold Nanoclusters for Cancer Radiation Therapy. Adv. Healthc. Mater..

[86] Chattopadhyay N., Cai Z., Kwon Y.L., Lechtman E., Pignol J.-P., Reilly R.M. (2012). Molecularly targeted gold nanoparticles enhance the radiation response of breast cancer cells and tumor xenografts to X-radiation. Breast Cancer Res. Treat..

[87] Butterworth K.T., Coulter J., Jain S., Forker J., McMahon S., Schettino G., Prise K., Currell F., Hirst D.G. (2010). Evaluation of cytotoxicity and radiation enhancement using 1.9 nm gold particles: Potential application for cancer therapy. Nanotechnology.

[88] Wolfe T., Chatterjee D., Lee J., Grant J.D., Bhattarai S., Tailor R., Goodrich G., Nicolucci P., Krishnan S. (2015). Targeted gold nanoparticles enhance sensitization of prostate tumors to megavoltage radiation therapy in vivo. Nanomed. Nanotechnol. Biol. Med..

[89] Murphy C. How Can You Calculate How Many Atoms Are in a Nanoparticle?. http://sustainable-nano.com/2016/07/28/how-many-atoms-are-in-a-nanoparticle/.

[90] Chang M.-Y., Shiau A.-L., Chen Y.-H., Chang C.-J., Chen H.H.-W., Wu C.-L. (2008). Increased apoptotic potential and dose-enhancing effect of gold nanoparticles in combination with single-dose clinical electron beams on tumor-bearing mice. Cancer Sci..

[91] Chen N., Yang W., Bao Y., Xu H., Qin S., Tu Y. (2015). BSA capped Au nanoparticle as an efficient sensitizer for glioblastoma tumor radiation therapy. RSC Adv..

[92] Chithrani D.B., Jelveh S., Jalali F., Van Prooijen M., Allen C., Bristow R., Hill R.P., Jaffray D. (2010). Gold Nanoparticles as Radiation Sensitizers in Cancer Therapy. Radiat. Res..

[93] Coulter J.A., Jain S., Butterworth K.T., Taggart L., Dickson G., McMahon S.J., Hyland W.B., Muir M.F., Trainor C., Hounsell A. (2012). Cell type-dependent uptake, localization, and cytotoxicity of 1.9 nm gold nanoparticles. Int. J. Nanomed..

[94] Cui L., Tse K., Zahedi P., Harding S.M., Zafarana G., Jaffray D.A., Bristow R.G., Allen C. (2014). Hypoxia and Cellular Localization Influence the Radiosensitizing Effect of Gold Nanoparticles (AuNPs) in Breast Cancer Cells. Radiat. Res..

[95] Geng F., Song K., Xing J.Z., Yuan C., Yan S., Yang Q., Chen J., Kong B. (2011). Thio-glucose bound gold nanoparticles enhance radio-cytotoxic targeting of ovarian cancer. Nanotechnology.

[96] Jain S., Coulter J.A., Butterworth K., Hounsell A.R., McMahon S., Hyland W.B., Muir M.F., Dickson G.R., Prise K., Currell F. (2014). Gold nanoparticle cellular uptake, toxicity and radiosensitisation in hypoxic conditions. Radiother. Oncol..

[97] Jain S., Coulter J., Hounsell A.R., Butterworth K., McMahon S., Hyland W.B., Muir M.F., Dickson G.R., Prise K., Currell F. (2011). Cell-Specific Radiosensitization by Gold Nanoparticles at Megavoltage Radiation Energies. Int. J. Radiat. Oncol..

[98] Joh D.Y., Sun L., Stangl M., Al Zaki A., Murty S., Santoiemma P.P., Davis J.J., Baumann B., Alonso-Basanta M., Bhang D. (2013). Selective Targeting of Brain Tumors with Gold Nanoparticle-Induced Radiosensitization. PLoS ONE.

[99] Kaur H., Pujari G., Semwal M.K., Sarma A., Avasthi D.K. (2013). In vitro studies on radiosensitization effect of glucose capped gold nanoparticles in photon and ion irradiation of HeLa cells. Nucl. Instrum. Methods Phys. Res. Sect. B Beam Interact. Mater. Atoms.

[100] Khoshgard K., Hashemi B., Arbabi A., Rasaee M.J., Soleimani M. (2014). Radiosensitization effect of folate-conjugated gold nanoparticles on HeLa cancer cells under orthovoltage superficial radiotherapy techniques. Phys. Med. Biol..

[101] Kong T., Zeng J., Wang X., Yang X., Yang J., McQuarrie S., McEwan A., Roa W., Chen J., Xing J.Z. (2008). Enhancement of Radiation Cytotoxicity in Breast-Cancer Cells by Localized Attachment of Gold Nanoparticles. Small.

[102] Liu C.-J., Wang C.-H., Chen S.-T., Chen H.-H., Leng W.-H., Chien C.-C., Wang C.-L., Kempson I., Hwu Y., Lai T.-C. (2010). Enhancement of cell radiation sensitivity by pegylated gold nanoparticles. Phys. Med. Biol..

[103] Liu C.-J., Wang C.-H., Chien C.-C., Yang T.-Y., Chen S.-T., Leng W.-H., Lee C.-F., Lee K.-H., Hwu Y., Lee Y.-C. (2008). Enhanced X-ray irradiation-induced cancer cell damage by gold nanoparticles treated by a new synthesis method of polyethylene glycol modification. Nanotechnology.

[104] Liu Y., Liu X., Jin X., He P., Zheng X., Dai Z., Ye F., Zhao T., Chen W., Li Q. (2015). The dependence of radiation enhancement effect on the concentration of gold nanoparticles exposed to low- and high-LET radiations. Phys. Med..

[105] Rahman W.N., Bishara N., Ackerly T., He C.F., Jackson P., Wong C., Davidson R., Geso M. (2009). Enhancement of radiation effects by gold nanoparticles for superficial radiation therapy. Nanomed. Nanotechnol. Biol. Med..

[106] Taggart L.E., McMahon S.J., Currell F.J., Prise K.M., Butterworth K.T. (2014). The role of mitochondrial function in gold nanoparticle mediated radiosensitisation. Cancer Nanotechnol..

[107] Wang C., Li X., Wang Y., Liu Z., Fu L., Hu L. (2013). Enhancement of radiation effect and increase of apoptosis in lung cancer cells by thio-glucose-bound gold nanoparticles at megavoltage radiation energies. J. Nanoparticle Res..

[108] Wang C.-H., Zhang S.-Y., Fang Q., Shen Z.-J., Fan Z.-J., Jin X.-F., Zeng Y., Liu Z.-Y., Xie H.-Z. (2015). Renal Dysfunction and hsCRP Predict Long-term Outcomes of Percutaneous Coronary Intervention in Acute Myocardial Infarction. Am. J. Med. Sci..

[109] Zhang X., Xing J.Z., Chen J., Ko L., Amanie J., Gulavita S., Pervez N., Yee D., Moore R., Roa W. (2008). Enhanced radiation sensitivity in prostate cancer by gold-nanoparticles. Clin. Investig. Med..

[110] Roa W., Zhang X., Guo L., Shaw A., Hu X., Xiong Y., Gulavita S., Patel S., Sun X., Chen J. (2009). Gold nanoparticle sensitize radiotherapy of prostate cancer cells by regulation of the cell cycle. Nanotechnology.

[111] Zhang X.D., Wu D., Shen X., Chen J., Sun Y.M., Liu P.X., Liang X.J. (2012). Size-dependent radiosensitization of PEG-coated gold nanoparticles for cancer radiation therapy. Biomaterials.

[112] Hainfeld J.F., Dilmanian F.A., Zhong Z., Slatkin D.N., Kalef-Ezra J.A., Smilowitz H.M. (2010). Gold nanoparticles enhance the radiation therapy of a murine squamous cell carcinoma. Phys. Med. Biol..

[113] Jayarathna S., Ahmed F., O’Ryan L., Moktan H., Cui Y., Cho S.H. (2021). Characterization of a Pixelated Cadmium Telluride Detector System Using a Polychromatic X-ray Source and Gold Nanoparticle-Loaded Phantoms for Benchtop X-ray Fluorescence Imaging. IEEE Access.

[114] Manohar N., Reynoso F.J., Diagaradjane P., Krishnan S., Cho S.H. (2016). Quantitative imaging of gold nanoparticle distribution in a tumor-bearing mouse using benchtop X-ray fluorescence computed tomography. Sci. Rep..

[115] Nie X., Chen Y., Meng L.-J. (2019). Towards Zero Compton Background in X-ray Fluorescence Computed Tomography (XFCT). J. Nucl. Med..

[116] Lechtman E., Chattopadhyay N., Cai Z., Mashouf S., Reilly R., Pignol J.P. (2011). Implications on clinical scenario of gold nanoparticle radiosensitization in regards to photon energy, nanoparticle size, concentration and location. Phys. Med. Biol..

[117] Behrouzkia Z., Zohdiaghdam R., Khalkhali H.R., Mousavi F. (2019). Evaluation of Gold Nanoparticle Size Effect on Dose Enhancement Factor in Megavoltage Beam Radiotherapy Using MAGICA Polymer Gel Dosimeter. J. Biomed. Phys. Eng..

[118] Mlinarić A., Horvat M., Smolčić V.Š. (2017). Dealing with the positive publication bias: Why you should really publish your negative results. Biochem. Med..

[119] Kempson I. (2021). Mechanisms of nanoparticle radiosensitization. Wiley Interdiscip. Rev. Nanomed. Nanobiotechnol..

[120] Shrestha S., Cooper L.N., Andreev O.A., Reshetnyak Y.K., Antosh M.P. (2016). Gold Nanoparticles for Radiation Enhancement in vivo. Jacobs J. Radiat. Oncol..

[121] Mackey M.A., El-Sayed M.A. (2014). Chemosensitization of Cancer Cells via Gold Nanoparticle-Induced Cell Cycle Regulation. Photochem. Photobiol..

[122] Li Q., Huang C., Liu L., Hu R., Qu J. (2018). Effect of Surface Coating of Gold Nanoparticles on Cytotoxicity and Cell Cycle Progression. Nanomaterials.

[123] Hanžić N., Horvat A., Bibić J., Unfried K., Jurkin T., Dražić G., Marijanović I., Slade N., Gotić M. (2018). Syntheses of gold nanoparticles and their impact on the cell cycle in breast cancer cells subjected to megavoltage X-ray irradiation. Mater. Sci. Eng. C.

